# “T*here’s no us vs. them, it’s just us*”: a creative approach to centring lived experience within the AVATAR2 trial

**DOI:** 10.1186/s12888-024-06268-z

**Published:** 2024-11-15

**Authors:** Oliver Owrid, Leonie Richardson, Stephanie Allan, Amy Grant, Sarah Gogan, Nicholas Hamilton, Francis Yanga, Nicola Sirey, Suzy Syrett, Philippa Garety, Tom Craig, Moya Clancy, Vanessa Pinfold, Lucy Miller, Thomas Ward, Clementine Edwards

**Affiliations:** 1 Department of Psychology, Institute of Psychiatry, King’s College London, Psychology & Neuroscience, London, UK; 2 South London & Maudsley NHS Foundation Trust, London, UK; 3 University of Glasgow, Glasgow, UK; 4 NHS Greater Glasgow & Clyde, Glasgow, UK; 5 The AVATAR2 PPI Group, London, UK; 6 NHS Research Scotland Mental Health Network, Glasgow, UK; 7 The McPin Foundation, London, UK

**Keywords:** Lived experience, Patient Public Involvement (PPI), Service user, Mental health, Research, Involvement, Creative, Co-production

## Abstract

This article describes a Patient and Public Involvement (PPI) led creative workshop space held within a clinical trial of a talking therapy for distressing voices (AVATAR2). PPI adds significant value to clinical research and ensures the work is meaningful to patients and their supporters. However, known issues include tokenism, a common power imbalance between PPI colleagues and researchers and a lack of opportunity for PPI to shape the research. PPI has played a key role at all stages of the AVATAR2 trial, including design, recruitment of staff and participants, data collection, analysis, and dissemination. An active and creative group of people was established, with over 30 members flexibly involved across all four sites. PPI group members were from diverse backgrounds, with lived experience of mental health conditions and recovery, and including carers**.** During this work, PPI colleagues identified that they would value a creative space which would help to promote the study but extend beyond an exclusive focus on trial deliverables. A regular creative workshop was established to support PPI colleagues in their creative work, including material such as poetry, blogs, art and podcasts. PPI colleagues and trial staff have cowritten a reflective piece to share their experiences on the impact of the creative workshops, organised into four themes. We found the workshops to be a powerful tool for forging relationships among trial staff and PPI colleagues, while also fostering personal development. We reflected on how the workshops built up the confidence of attendees and supported demanding trial activities such as public speaking. Finally, we discussed the impact of the workshops on wider trial culture, by upholding the values of the team and challenging the status quo.

**Supplementary Information:**

The online version contains supplementary material available at 10.1186/s12888-024-06268-z.

## Background

Within healthcare, there has been growing inclusion of the expertise acquired through lived experience of mental health conditions or differences. Within research, this takes the form of projects that are done ‘with’ or ‘by’ healthcare service users as opposed to ‘about’, ‘to’ or ‘for’ them. In the most extended form, there are user-controlled or survivor research projects, which refer to research projects which are exclusively led by individuals with lived experience [1]. Another approach is co-production, where lived experience colleagues and conventional project leaders equitably share power, responsibility and remuneration. A more common approach in the UK is termed Patient and Public Involvement (PPI), in which project leaders often hail from professional research backgrounds and individuals with lived experience are employed as consultants. Arguments for the inclusion of lived experience within research range from efficiency-oriented to moral [2]. It is widely believed that the inclusion of experiential expertise results in research which is more relevant, accessible and helpful for the populations that it concerns [3], while further arguments describe a moral duty for researchers to share power about deciding which clinical issues are prioritised in research innovation and funding [4]. These benefits are arguably best exemplified in projects with greater degrees of lived experience involvement. In recent years, such benefits have been deemed essential. Evidence of robust and meaningful PPI has, therefore, become a funding requirement and key strategy component for many major funders, such as the National Institute of Health Research (NIHR) and Wellcome [5].

Despite increasingly supportive policy from key funders, there are known barriers to the implementation of PPI within mental health research. A key concern expressed by some researchers and funders is that PPI may result in increased research time and costs [6]. With PPI as a funding requirement, plans for involvement may also risk becoming tokenistic or a tick-box exercise, and PPI has often been criticised for fitting within a system that marginalises lived experience voices as opposed to promoting radical change [7]. Several co-authors of this paper report experiences of tokenism during past research involvement, echoing views expressed across the wider literature [8, 9]. Further challenges include the emotional labour of bringing personal experiences to research settings, marked inequalities between researchers and their PPI colleagues and an inadequate focus on relationship building [10, 11]. These issues prevent people with lived experience of mental health problems (and their carers and supporters) from participating in PPI work in both the short term, by creating barriers to participation, and in the longer term, by making PPI colleagues walk away. Taken together, these issues and tensions present substantial barriers to the implementation of PPI within mental health research.

Initiatives to address these issues are ongoing and take multiple forms. The NIHR has developed six value-based standards to improve the quality and consistency of public involvement. The standards emphasise inclusive opportunities, working together, support and learning, communication, and impact and governance. A growing body of researchers have now translated these standards into projects and provide additional learning through sharing their positive experiences of PPI collaboration [11, 12]. Efforts to ensure that PPI opportunities are more than a ‘tickbox exercise’ rely in part on the policy of funding organisations. It is now explicitly stated by funders that PPI opportunities must be meaningful [13], a principle supported by the inclusion of individuals with lived experience within funding panels and governance bodies. Various user-controlled research projects have shared experiential learning around the emotional labour of PPI work and offer recommendations for best practice [12]. A fundamental recommendation is that the individual needs of each PPI group need to be listened to in order for relevant, personalised adjustments to be made [2, 11]. Common areas of adjustments include investment in relationships between researchers and PPI colleagues, as well as the provision of flexible and creative spaces for the discussion of lived experience [10, 11, 14]. In the present paper, we share our experiences of one such creative space, which emerged organically during PPI involvement within the recent AVATAR2 trial. AVATAR2 was the first multi-site randomised controlled trial of AVATAR therapy, a psychological intervention for distressing voices in which people engage in empowerment-focused dialogues with an “avatar” representing the voice [15]. The aim of this paper is to bring together reflections from across the team on the cascade of positive effects associated with the creative group. We share what we have learnt with the hope of encouraging others to consider ways of embedding creativity within PPI.

## Methods

### PPI within AVATAR2

The AVATAR2 trial took place between December 2019 and October 2023 and comprised four sites across London, Manchester and Glasgow. Throughout the trial, there were regular consultations with The McPin Foundation, a mental health research charity who provided expert advice on the setup and implementation of PPI in the trial. In line with this advice, each site formed a working group of individuals with lived experience relating to psychosis, who then contributed as PPI Consultants on the trial. PPI colleagues from across sites met with researchers regularly and contributed towards many key trial activities such as recruitment presentations to clinical teams and interview panels for the recruitment of trial staff. PPI colleagues were paid for their time in line with NIHR INVOLVE guidance [16], were invited to team meetings and were directly involved in conducting primary qualitative research to understand participant experiences of the therapy and wider trial procedures. Across the UK, PPI colleagues also crucially contributed to raising trial visibility through presenting posters and talks at conferences (King’s College London & NHS Research Scotland), giving interviews for national TV, a Wellcome campaign and on podcasts (BBC Scotland & Mental Elf). Each PPI colleague was paired with a Research Assistant (RA), and together they built a working relationship, with the RA acting as the main point of contact with the trial. These individual relationships were tailored flexibly to the needs of each PPI colleague. Some chose to receive just essential updates and meeting invites, whilst others valued greater involvement, which included co-producing personal development plans, receiving signposting to further work opportunities and meeting for regular catch ups over coffee.

### AVATAR2 PPI creative workshop programme

The AVATAR2 study website hosted a variety of blogs written by PPI colleagues and trial staff. Early in the trial, PPI-written blogs were conceived as a helpful resource for engaging potential teams and participants by providing first person accounts of therapy and demonstrating the extent of lived experience involvement within the trial. In order to support PPI colleagues with their writing, a single workshop was set up by two RAs, which focused on blog-writing techniques. This workshop was met with enthusiasm from attendees and led to a surge in written creative pieces. The trial’s leadership greatly valued PPI so following the request of PPI colleagues, workshops became a regular monthly meeting. The workshops were intended as a confidential and supportive environment for sharing, whether personally during the check-in or creatively during the sharing space. The content of the workshops was decided by PPI colleagues and over the course of the trial, expanded to include blogging, poetry, creative writing, podcasting, poster-making and spoken word. In the supplementary material of this paper, there are several exemplary creative pieces from the workshop program, along with a link to the co-produced AVATAR2 podcast.

**Fig. 1:**
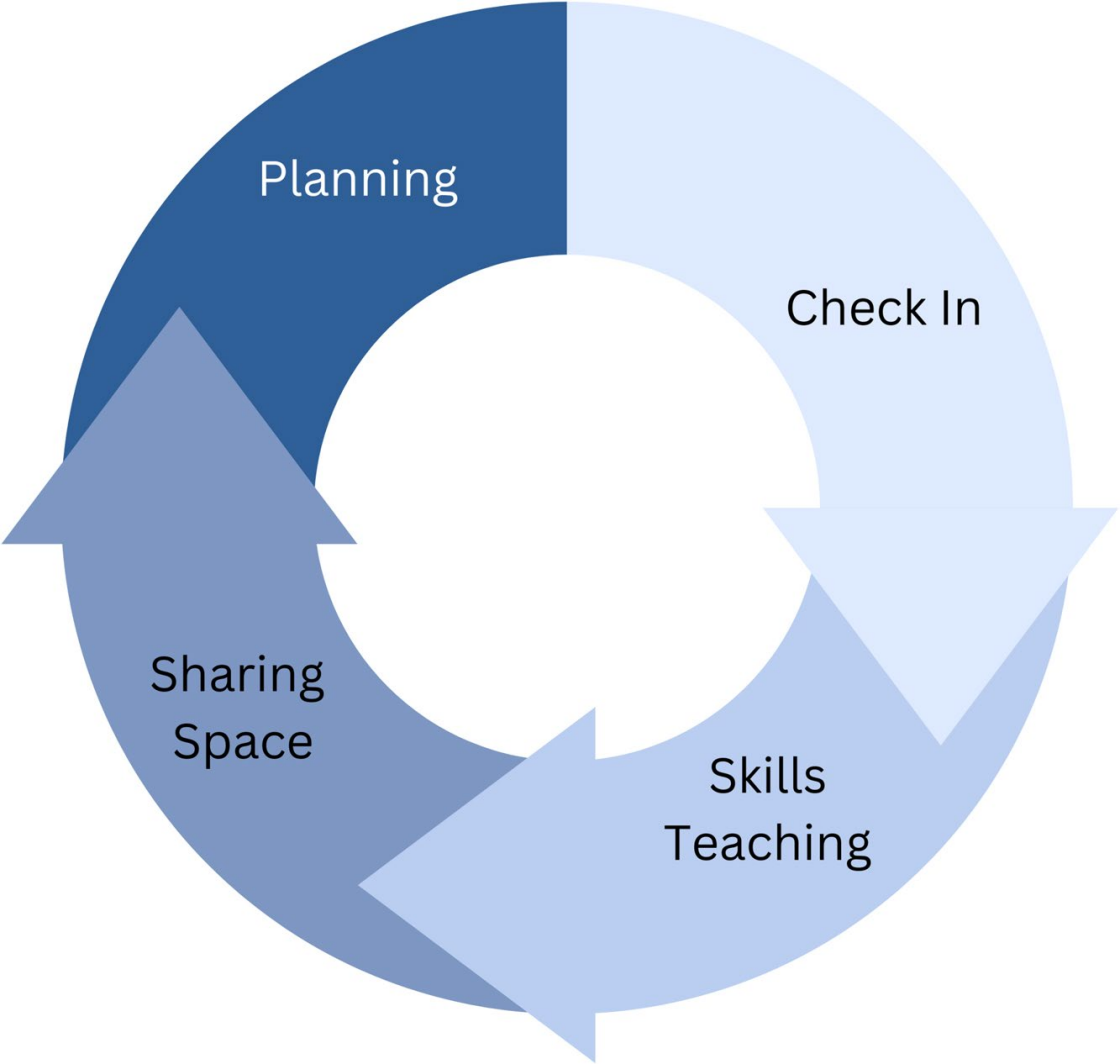
Flow diagram showing the structure of the creative workshop

The workshops lasted one hour, were hosted online to connect members who were geographically distanced from each other and held at a sociable hour on a Friday to help create a more relaxed atmosphere. Contrary to other meetings, attendance was wholly voluntary and unpaid, however PPI colleagues received a flat rate (£50) for any trial-related blogs published on the website, to compensate for time spent writing. Workshops were facilitated by an RA who encouraged a warm, informal and interactive atmosphere. The RAs had not undergone clinical training, however had an undergraduate knowledge of Psychology, strong interpersonal skills and received weekly supervision with a Clinical Psychologist. At the beginning of each workshop, the facilitator (RA) offered an update about themselves, which would generally be detailed and open, before inviting other attendees to do the same if they wished. Check-ins were followed by a skills-focussed presentation, where the facilitator shared information about a creative topic the group had expressed an interest in (e.g. creative writing). Afterwards, there would be a sharing space, where attendees were invited to share current pieces of work for positive, constructive feedback from the rest of the group. Finally, the group would decide a date and focus for the RA to prepare for the next session. The structure of workshops is depicted as a flowchart in Fig. 1.

### Reflecting on the workshops

The present paper was co-authored by members of the AVATAR2 trial, led by RAs and PPI colleagues and supported by Trial Managers. The content is derived from what was shared in two reflective spaces where we discussed the impact of the creative workshops. PPI colleagues and RAs who had participated in the workshops were invited to a recorded meeting to reflect on the impact of the creative workshops from their first-hand perspective. Those who were unable to attend were invited to provide input on the written draft to provide an equitable co-authorship opportunity and control of manuscript content. Following this, all colleagues involved in trial management were invited to a session to share their reflections on the wider impact of the workshops. This included Principal Investigators, Trial Coordinators and Therapy Coordinators (responsible for training and managing therapists within the trial). The attendance of both spaces is detailed in Table 1. Both reflective spaces were co-facilitated by an RA and PPI Consultant, and all PPI attendees were paid for their time at NIHR involvement rates. For brevity, the first reflective space will be referred to as the Workshop Members (WM) Space’ and the second reflective session will be referred to as the ‘Trial managers (TM) space’.

**Table 1:** Composition of reflective spaces

	**Role**
**Workshop Members**	PPI Consultant
**Reflective Space**	PPI Consultant
	PPI Consultant (Co-facilitator)
	Research Assistant (Co-facilitator)
	Research Assistant
	Research Assistant^a^
	Research Assistant
	Lived Experience Researcher
**Trial Managers**	Trial Manager
**Reflective Space**	Trial Manager
	Therapy Coordinator
	Therapy Coordinator
	Principal Investigator
	Co-Principal Investigator
	Research Assistant (Co-facilitator)
	PPI Consultant (Co-facilitator)

^a^With a schizophrenia diagnosis and previous experiences as a PPI Consultant

For analysis, we have not employed a formal qualitative methodology. The decision behind this was to increase the accessibility of the research process, in line with a co-produced ethos. It has been noted that research which claims to be participatory can actually be co-opted by researchers who get to set the agenda and decide what findings are meaningful [8]. To challenge this, we met together as a team in discussion spaces where everyone could contribute what they wanted to include in the paper and what they felt were important reflections to share. These reflections have been summarised as key learning themes with supporting quotes from the discussion we had for transparency. Consent was obtained in the decision to be a co-author. All co-authors were informed that this would involve sharing their views and/or editing the paper alongside known trial collaborators. All co-authors were given final control over the content of the manuscript, including selection of quotes.

**Fig. 2:**
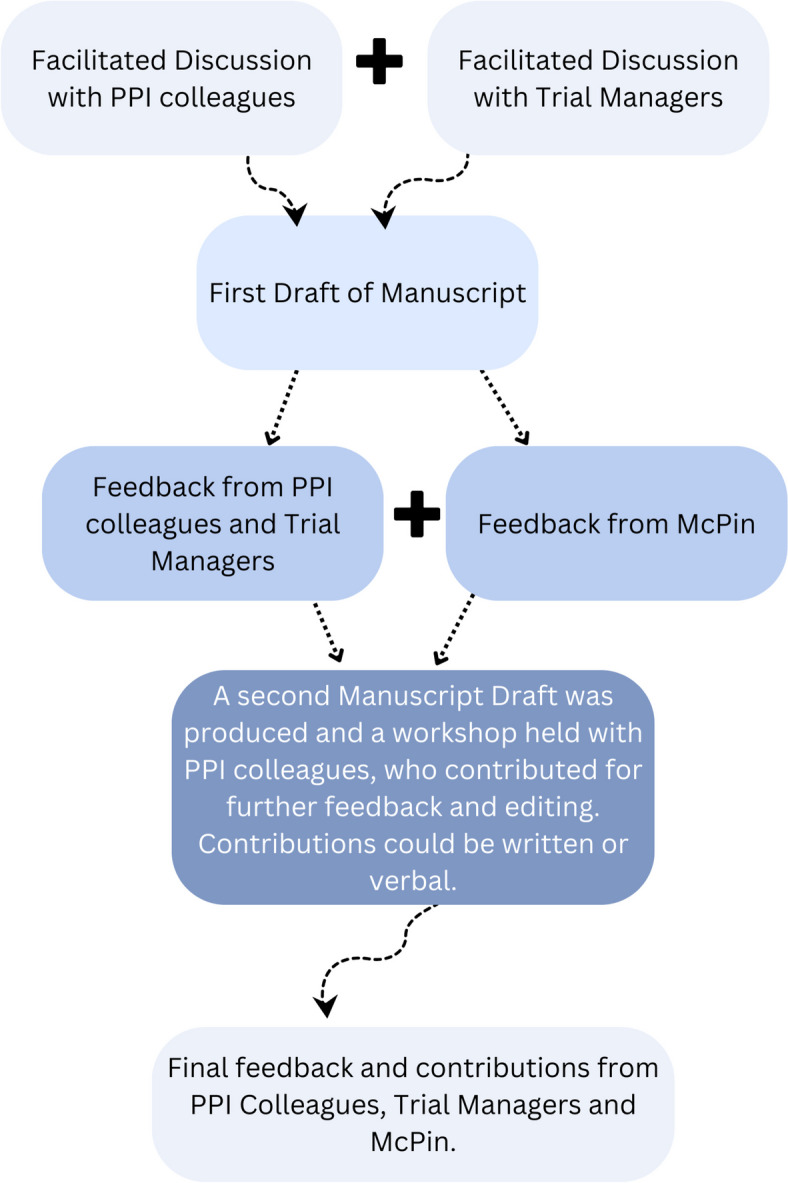
Flow diagram showing the progression of the manuscript and the co-writing process

Transcriptions of the workshops were obtained from Microsoft Teams. Three Research Assistants (OO, LR and SA) read the transcripts, independently organised the content into initial themes and extracted salient quotes from the discussions. These authors then triangulated their interpretations of the content together and agreed a set of themes. A summary of these themes and transcripts were then shared with attendees of both reflective spaces, with an invitation to make changes. Feedback was also sought from McPin, for guidance on the background and methodology of the manuscript. Following this, a half-day workshop was held to collectively edit the paper in real-time with PPI colleagues. The latter involved both ‘live’ editing and rewriting some sections as a group, as well as some discussions around ideas PPI colleagues saw as missing from the initial draft, e.g. the impact of publishing creative work on the trial website. RAs then incorporated these changes in an updated version of the paper. The process is detailed in Fig. 2. For clarity, we have indicated throughout the paper which reflective space initial ideas originate from. Where a perspective importantly relates to a co-author’s role, we have also detailed this. Exemplar quotes from these reflective spaces are used throughout to ensure that voices are represented.

## Results

Overall, there were 15 workshops held between December 2021 and June 2023. These were attended by 18 different individuals overall (median number of attendees = 6), which consisted of 11 PPI colleagues and 7 trial staff.

### Key thematic reflections

**Fig. 3:**
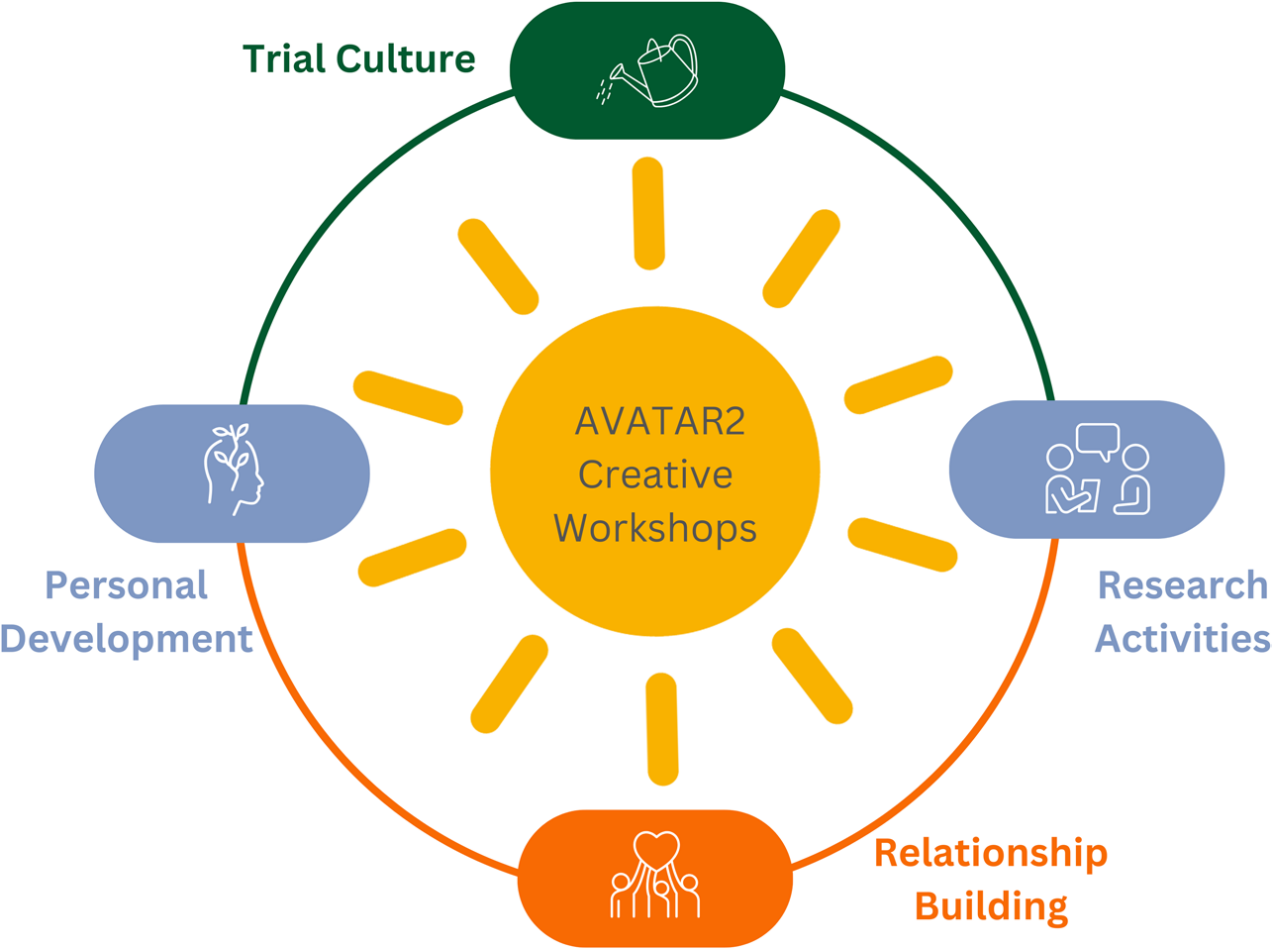
Diagram illustrating the main themes drawn from the two reflective spaces. This is conceptualised as bottom-up (relational foundations) and top-down (over-arching culture) which came together to facilitate benefits on personal and practical levels

We organised our key reflections into four main themes (Fig. 3): Relationship Building, Personal Development, Research Activities and Trial Culture.

### Relationship building

The workshops started in the second year of the project, and as such there were pre-existing relationships between some PPI colleagues and RAs, however a key reflection was on how these relationships developed through the workshops. The quality of relationships formed in the creative workshop setting was commonly contrasted with previous interactions in other research projects. These previous interactions were felt by PPI colleagues to be entirely one-sided, consisting of researchers expecting PPI colleagues to share their opinions and deeply personal experiences of mental health problems with little reciprocation or even warmth. This is exemplified in this reflection:

*“So obviously I got colder because they [staff from a previous research study] wouldn't warm up to me back, so it was a bit awkward. So I was really open and they were really closed”.—*PPI Consultant (WM Space).

One-sided interactions were felt to entrench a clear division and power imbalance between those who consider themselves as ‘the real researchers’ and those they see as the lived experience consultant. To be on the receiving end of this has felt akin to being in a “perpetual interview”, with the lack of warmth and reciprocation ultimately closing off opportunities for an enjoyable working relationship. Over time, the accumulation of these experiences was described as demotivating and led some of our PPI colleagues to even want to quit the field of mental health research altogether:

*“I almost quit doing PPI work I think a few years ago, because of the feeling of constantly feeling like a patient, I'm not really feeling like on the same level as the people that I'm working with because I consider you a colleague, but they don't consider me a colleague and it was kind of hurtful, even though they didn't say it.”—*PPI Consultant (WM Space).

The creative workshop space helped to purposefully develop relationships which were different from those described above as the focus was intrinsically reciprocal with everyone sharing experiences. In the WM Space, we reflected that the workshops offered a space to connect on a more equal and human level and the ‘check in’ section was key to this. While check-ins could take any tone, often they had a quality of humour and were encouraged to be reflective and non-judgmental. This segment helped to set the tone of sharing, authenticity and familiarity for the meeting and sometimes took over 30 min, which helped develop positive relationships.

Within the WM Space, we also spoke about how the personal focus of the work (whether produced by an RA or PPI colleague) would lead to broad discussions that spanned personal experiences of mental health problems, as well as discrimination, art, poetry and relationships. These discussions were felt to be deeply connecting and inspired attendees to produce creative pieces, some of which were shared on the trial website. This overall positive change in relationship development is exemplified in the following reflection which highlights how in the workshop we were all on the same team, regardless of whether someone came from a research or lived experience background (or both). In other words:

“*there’s no us vs. them, it’s just us*”—PPI Consultant (WM Space).

### Personal development

Across both spaces, we reflected on the beneficial impact of the creative workshops on the personal development of team members. From all perspectives, the growth in confidence of PPI colleagues was highlighted. In the WM Space, several PPI colleagues spoke about their previous negative experiences of work environments, which included experiencing bullying and discrimination linked to their lived experience status. We discussed how having a warm, positive working environment with spaces like the creative workshop countered these memories and created hopeful prospects for future work opportunities. In particular, the workshops helped to build confidence in public speaking and creative ability:

*“It gave me confidence, no way on Earth [before this] could I do a panel presentation. I had no clue I could put a poem together. It really helped me as I could write about challenges of voices and paranoia that I’d never done before and didn’t know I could do. In terms of how they’ve built my confidence, it’s blown me away.*”—PPI Consultant (WM Space).

Within the TM Space, the creative workshops were discussed as a welcomed opportunity to explore different ways of working and support PPI colleagues to grow their talents and skills. Within both spaces, we reflected on the evolution of PPI-created poetry within the trial, the performances of which became a celebrated feature of our bi-annual whole team meetings:

“*It’s been lovely to *w*atch [PPI Consultant]’s confidence grow and see it flourish as well because I know at the very beginning you were maybe feeling like a bit unconfident to share one of the pieces, to you posting several of your poems on the blog and reading them out yourself. It’s been amazing to see.”* Research Assistant (WM Space).

The growth in confidence was experienced as deeper than just trial activities and described by one PPI colleague as ‘developing my voice’. Some PPI colleagues have since taken their enthusiasm beyond the trial and have submitted original poetry in competitions, attended poetry recitals and engaged with creative writers’ workshops in other settings. During the WM Space, we also spoke about the impact of having creative pieces published on the trial website, which was felt to show the importance of the PPI perspective. On a practical level, this also provided PPI colleagues the opportunity to cite their work in applications to future employers, which was validating.

Within the WM Space we discussed how, in addition to developing skills, the atmosphere of workshops and process of sharing seemed to offer benefits of a therapeutic quality. Workshops provided a rare space to speak about issues of stigma and discrimination within a warm and confidential setting, as exemplified in the quote below.

*“I think what it stands for is our own personal understanding of ourselves and our ability to share and articulate what it is we're feeling and maybe have some understanding of the context of why we're feeling that … it's really been a privilege to be part of this group because hearing people express often very painful experiences in such an open and articulate and unafraid way has been something I've certainly valued and come away from feeling, like, less alone.”—*Lived Experience Researcher (WM Space).

The monthly recurrence of workshops provided a helpful structure and accountability to produce creative work, which helped attendees to set goals and make plans over the course of the months. We discussed how this was particularly helpful for attendees with difficulties in self-motivation, as it pulled focus towards the future as opposed to the past.

## Research activities

Workshops were initially conceived to support PPI colleagues with writing blogs for the trial website, and within the TM Space we discussed their positive influence across this and a range of other research activities. From the trial delivery perspective, it was important that recruitment was achieved to target and on time despite taking place during the COVID-19 pandemic, with high numbers of participants remaining engaged throughout the duration of the trial. We discussed how the activities of PPI colleagues were crucial in meeting this goal. Since the introduction of workshops, we have benefited from a diverse and consistent collection of PPI-created content published on the website. Written pieces from PPI colleagues provided first hand insight into experiences of the therapy *[*https://www.avatartherapytrial.com/post/experiencing-avatar-therapy*]* and communicated the ethos of lived experience involvement within the trial, which helped to assuage anxieties about research participation:

*“Definitely there is the impact on recruitment, engagement in therapy and therapy completion rates. I think there's something about knowing that there is a group of lived experience people who are providing materials that we can draw on and share with potential participants. Or that if someone clicks on our website, we know that they're going to access materials in different and innovative ways, that means that we can feel confident when we recommend it.”*—Trial Manager (TM Space).

Within the WM Space we discussed how the creative skills taught in workshops were practical and helpful toward PPI activities, such as public speaking to clinical teams. PPI colleagues also felt that the confidence gained through workshops had increased their willingness to share their ideas in team settings. This shift was acknowledged in the TM space, where Trial Managers noted that since the introduction of the workshops, attendees of the workshops had been more confident in expressing their thoughts and ideas during wider team meetings, leading to a richer quality of consultation:

*“I've known people who are involved in the workshop for quite a number of years and just seeing people flourishing and engaging with something and presenting and putting themselves and their work and their ideas out there. It’s been remarkable.”*—Therapy Coordinator (TM Space).

Within the TM Space, we remarked upon the emotional demands of a wide breadth of involvement of lived experience within the AVATAR2 trial. We discussed how working in this way required a level of emotional support, which appeared to be provided in part by the creative workshops. Anxiety-inducing experiences such as public speaking might in some contexts feel negative, however with the right support they turn into valuable growth opportunities. This seemed to create a virtuous circle within the AVATAR2 trial, with increasing adventurousness around the scope of PPI activities as the trial progressed. This culminated in the AVATAR2 podcast [https://www.avatartherapytrial.com/resources?wix-music-track-id=9711878123451216]*,* where PPI colleagues spoke about their experiences of working within research and performed original-written poetry.

*“Knowing that we're providing the extra support and space for people to think about their experiences and express themselves, I think, meant that when people did contribute to recruitment presentations and things you kind of could tell that they've been accessing that extra support, and I think more people would then grow in confidence and feel more able to help with other things.”—*Trial Manager (TM Space).

### Trial culture

Within both spaces we discussed the ethos of inclusivity and coproduction which we experienced within the trial. We felt that this was the bedrock for positive working relationships and successful PPI work. The creative workshops were seen as being both a product and driver of this accommodating atmosphere:

*“I felt like it's* [the creative workshops] *created a space where because everything is so coproduced and everyone's involved it, it seems to have just really helped everyone grow in confidence in these working relationships”—*Trial Manager (TM Space).

In the TM Space, we discussed how one of the impacts of the workshops on trial culture was through the introduction of creative practices into trial spaces. We felt that these workshops demonstrated a novel, broad and peer-led approach to PPI, which challenged our pre-conceptions and pushed the boundaries of lived experience involvement. A key lesson that we had taken away was the importance of a willingness to learn from the unexpected. Colleagues with experience managing previous trials reflected how some of the most meaningful and formative PPI contributions occurred organically and were not set out in advance:

*“In the [previous] trial we had PPI and one of the things that the PPI group said, looking at the impact at the end, that they thought was most important was the unplanned and unanticipated things that the PPI group did. And when we set up research we often have to do everything in a very planned way.*—Principal Investigator (TM Space).

Within the TM Space, we discussed how the speeches and performances given by PPI colleagues during biannual whole team meetings were experienced as particularly impactful on our trial culture. The content of each PPI-members' presentations was always entirely peer-led, supported by the RAs, and included pre-recorded and live readings of creative works. Platforming of first-hand lived experience voices in these settings made the research itself feel more real, engaging and moving across team members. This was an opportunity to engage with the core values and approach of AVATAR therapy and the trial team, with a strong focus on empowerment and social inclusion. Bringing these values into focus at whole team meetings fostered motivation and connectedness across the geographically scattered team:

*‘The wider team has come away feeling the work that we're doing in this area of work is motivating. So I think you've had an impact of really motivating the wider team and that's the kind of interesting way of looking at the sort of PPI that you've been the drivers of.’***—**Principal Investigator (TM Space).

In a reciprocal way, the culture of the trial inextricably influenced the tone of the workshops too. In the WM Space we discussed a felt sense of openness to PPI-led ideas throughout the team and a commitment to making them happen. This led to the formation of the creative workshop programme as well as the evolution and breadth of PPI activities from blogging to poetry and podcasting. This culture was felt to be possible through the industry of PPI colleagues and RAs, but also reflected the integrity and cohesion of the wider team towards centring lived experience. In the WM Space we felt that this culture communicated a sense of inclusion, connectedness, and the presence of shared values across the team:

*“There just seem to have been no boundaries and I like that. There’s this kind of ‘Yes Culture’, you know as long as none of us are asking anything really silly. It just feels really inclusive.”***—**PPI Consultant (WM Space).

## Discussion

In this co-written paper we have shared learnings from a creative workshop which helped to shape the culture of a clinical trial. We have found that a shared creative space enabled us to move beyond “research outputs” and “deliverables”, and foreground opportunities to connect and be human together. PPI colleagues reflected that having a space like this within the AVATAR2 trial was a challenge to the usual status quo of PPI within clinical research and enabled people to develop and pursue their own interests in addition to contributing planned PPI inputs on the study. PPI has been critiqued for failing to radically challenge a system where patients seldom get to take up positions of power within research [17]. This PPI criticism echoes further concerns from peer support workers who feel relatively powerless and that their roles are devalued within the mental health system compared with other mental health staff [18].

The workshop helped to facilitate more reciprocal relationships, thereby addressing the known risk of power imbalance in PPI and other peer roles. The approach is consistent with growing calls to champion ways in which PPI colleagues can participate in knowledge generation, without being discounted when their chosen form of communication and expression does not fit what is typical within the system [19].

In sharing our learnings, we hope to inspire others to get involved meaningfully, and perhaps differently, with PPI work. We encourage researchers, and others who are planning for projects incorporating lived experience perspectives, to be open to challenging pre-conceptions they may have about what PPI work can or should look like and to build-in space for “unplanned” activities which foster relationships and creativity. While “unplanned” involvement opportunities will undoubtedly vary across different contexts we want to highlight the importance we found in shared team values of empowerment and social inclusion. These values underpinned our approach to PPI and helped build confidence in others to embrace the process of learning and building spaces, collaboratively.

For several colleagues, writing this paper was motivated by a desire to set a new standard for centring lived experience in research and improve the experience of others who wish to personally get involved with PPI work in future. We acknowledge the breadth of PPI activities across the AVATAR2 trial was enabled through sufficient funding to appropriately reimburse colleagues and adequate staff and resources in the trial team to support this work. The large scale of the project also allowed a critical mass of attendees at the workshop to develop which may take longer to achieve in smaller projects. Workshops themselves were unpaid as they were intended as an optional, supportive learning space as opposed to output-driven work. Furthermore, we wanted to provide equitable funding opportunities to PPI colleagues across the trial who would not be interested in the workshops, so chose to fund the production of creative pieces instead. Over the course of the AVATAR2 trial there has been a willingness to augment the budget originally allocated to PPI to support the flourishing of the PPI work. Researchers planning future studies and wishing to adopt this approach should consider this in funding applications, and funders must also encourage sufficient resourcing to enable meaningful lived experience involvement, with flexibility for innovative involvement plans that emerge during the study through collaboration.

Furthermore, we must recognise recent criticisms of PPI work in mental health research as contributing to ‘elite capture’ [19], whereby the expectations placed by researchers on how lived experience expertise can contribute to and is incorporated into research can exclude people from already marginalised groups. On the AVATAR2 trial, we adopted a flexible involvement model for PPI which recognised that people’s life circumstances, health, availability, and interest in PPI may change over time, affording people choice and control over their involvement. This, we believe, helped increase the inclusivity of the AVATAR2 PPI group. Limits to accessibility were also identified through the manuscript drafting process. For example, we found that within the co-author group there were different levels of confidence in engaging remotely with the manuscript presented in a traditional academic format. To overcome this barrier, we applied learning from the creative workshop format to facilitate a process that was open to all collaborators. This took the form of the manuscript editing workshop with RAs and PPI colleagues, where we collectively read through the draft manuscript and made changes in real time. We also recognise the importance of striving to engage a range of perspectives, reflecting the diverse and intersecting identities of people who experience psychosis. This is a challenge facing all researchers in psychosis, and we are committed to working to improve representation in this area.

Finally, the reflections in this paper reflect the views of our circumscribed working group. We acknowledge that the workshop format has resonated well *because* it was co-developed by us and perhaps this may not apply to other working groups. However contrary to this, several co-authors of this paper have since been approached by other research groups and organisations who, inspired by reading blogs on the trial website and listening to the Creative Workshop podcast, wish to reproduce similar spaces and projects in their own context. We believe this points to the wider relevance of the approach discussed in this paper. As a group, we feel proud of what was achieved within the AVATAR2 trial and to see some of these already cascading effects. To the best of our knowledge, the incorporation of a creative workshop into a clinical trial was novel. We hope this may encourage others to come up with new and creative approaches to involvement, which we hope to hear about in the future:

*“I just get a great sense of achievement for myself. And not only do I feel that our voices were heard, but that we are voices for other people in the future, for other PPI Consultants. And for them, if they read this, to be confident to be PPI Consultants themselves and get involved and make a difference, because I feel like we have made a difference.”—*PPI Consultant (WM Space).

### Limitations

This paper was conceptualised at the end of the trial, meaning we faced time and resource pressures which prevented us from offering team-wide training in qualitative approaches. We therefore decided to not use a formal qualitative methodology primarily to create an authentic co-authorship opportunity that did not restrict collaborators without professional research backgrounds. We accept that this may lessen the transferability of our approach and so took steps to address this through adopting a reflective stance, including a broad range of co-authors and a collaborative, iterative writing process.

In generating data, we held reflective spaces where attendees were simply asked to discuss their perceived impact of the workshops. This openness was intended to stimulate discussion, foster free association and gather dominant ideas. However, this setup meant that we did not gather reflections on areas that attendees did not think of or feel able to share in this setting. This contributed toward an at times imbalanced focus on PPI colleagues, for example with regards to the personal development. The personal development of RAs was comparatively under explored in these reflective spaces, despite being frequently discussed during the creative workshops themselves. We encourage further research which explores the impact of such programmes for wider colleagues.

## Conclusion

While PPI is commonly criticised for failing to make a radical impact on research, we found that with creative freedom and adequate financing for PPI colleague time, we were able to achieve a level of lived experience involvement which was meaningful and influential within this trial. Lived experience work requires a unique blend of professional and personal identity in a manner that other professional roles largely do not [20], and there are growing calls that this should be addressed and supported in the working environment [10]. Our creative workshop approach might be one way of meeting these needs, by offering a supportive space where relationships can be forged and issues of lived experience can be articulated without the confines or expectations of other research spaces. The relationships formed, skills developed, and confidence gained through workshops facilitated progressively ambitious forms of involvement and has left a lasting impact on attendees that extends beyond the AVATAR2 trial. We hope that the creative works produced, along with this article, show the impact that organic, peer-led initiatives can have and encourage others to approach PPI with creativity.

## Supplementary Information

Supplementary Material 1.

Supplementary Material 2.

## Data Availability

Within the supplementary material, we have included several examples of creative pieces produced within the creative workshops. The full catalogue of PPI-created content as part of AVATAR2 can be found on the trial website (avatartherapytrial.com/news-blog), which we highly recommend visiting. The reflective spaces were used as a space to share our ideas as opposed to collect data per se therefore we have not shared the transcripts, however we have included further exemplary quotes from these spaces in the supplementary material.

## Abbreviations

PPI: Patient public involvement
WM Space: Workshop members’ reflective space
TM Space: Trial managers’ reflective space
