# Supplementary material for: “There’s no us vs. them, it’s just us”: a creative approach to centring lived experience within the AVATAR2 trial
Source: BMC Psychiatry. 2024 Nov 15;24:807. doi: 10.1186/s12888-024-06268-z (PMC11567013; doi:10.1186/s12888-024-06268-z)
Supplement: Supplementary file 1 — Supplementary Material 1. [file 12888_2024_6268_MOESM1_ESM.docx]

**Supplementary Material 1: Creative Pieces**

**My Satellite**

Hope is a Moon.

It is always there.

You just experience different Moon phases.

Even when you can’t see it some days.

At times, it will eclipse everything.

Other days, it may feel like a waning crescent.

Maybe yours is always a beautiful full Moon.

It’s okay to feel like you’re down to your last quarter.

Your first quarter can be restored by a ‘Once in a blue moon’ moment.

Observing a new Moon.

Sometimes, we just need the right binoculars or telescope to experience the Moonlight.

*By Amy Grant*

**Garden of Words**

Feeling like a seed not allowed to become a tree.

Anxiety, the fear that befriended me.

Comfort zones became panic rooms to hide.

A bigger fear of missing out began to grow inside.

Overshadowed by self-doubt but found ways that illuminated.

I am the author, Anxiety just sometimes narrated.

When anxiety talks, Me; a radiant, ready reply...

"I won't know, until I try"

*By Amy Grant*

**My Mask**

I don’t want to paint on my mask today,

I want it all to go away.

I want life how it was before,

I’m not sure I can cope anymore.

Then the voice of hope creeps in my ear,

But I hit it away as it fills me with fear.

Hope means things may work out fine,

But this is such a very thin line;

Balanced on a tightrope that may break at any time.

I don’t want hope as it’s a lie,

I just really, really want to know why.

Yet my voice of reason is loud and clear

It speaks to me and comforts my fear.

This is just one day, and I can make it,

Even if I have to fake it.

Tomorrow I may feel better and strong,

Tomorrow I may sing my inner song,

Tomorrow applying my mask may not take so long.

*By Sarah Gogan*

**Descent**

Resting on the sofa I glimpse my daughter,

Head up, pondering the stars.

Contented in the moonlit courtyard, Kitty at her feet.

What does she see?

The Giant falling to Jack?

Rapunzel shorn and tumbling from the tower;

Or Sleeping Beauty spiralling down,

Awakening to her newly-kissed life?

Do fairy tales fill her head?

Perhaps, as she stands firm and strident,

An angel glides from the crescent moon,

Immersing her in buttery wings,

Soothing fears of what’s to come:

Other descents, in other futures.

But, here in the peace and quiet

I look up and see her ascend.

*By Sarah Gogan*

**Thought Broadcast**

Inserting thoughts into my brain

Thought deceivers

Suspicion

Irrational thoughts

I can’t escape, things escalate

What do I do with this information?

Forces me back to my lonely isolation

Please please don’t act on compulsion

It all don’t make sense, you will be full of repulsion

People won’t understand, they won’t show you compassion

Like their feelings, their empathy are all on ration

The thoughts aren’t mine, they’re on repetition

My downfall, my demise, my brains demolition.

*By Nicola Sirey*

**Shadow People**

Shadow people they can fly

LSD, no I’m not high

My dedication in taking medication

A precaution, to get rid of the distortion

My mind tied up in metal chains

The fear of the shadow people still remains

I have an aversion to the coercion

From the shadow people’s commands

My discipline to not give in to their demands

I tell you shadow people, I am not insane

My hopes of getting better still remain

You are not my friend, or my mate

I am strong and I will decide your fate

You are dark, you are mean, you are outrageous

I am brave, I am powerful, I am courageous.

*By Nicola Sirey*

**Life Since AVATAR Therapy**

I was a participant in the first AVATAR trial, which happened 5 years ago and it really helped to open so many new doors for me. This blog is all about my onward journey to hopefully getting completely better, as I suffer from psychosis. In this blog I am going to talk to you about the fact that I can now go to football matches, which I never thought would be conceivable in my wildest dreams.

I now go to watch Sutton United and Charlton Athletic play home and away games with my Dad and my eldest nephew. We have always been a really close family, but I now feel my bonds with them are even closer as we can share something special in supporting the same teams and the absolute joy when one of our teams wins a game. I know they say football fans are kind of tribal, and it is very true as sharing the absolute jubilation that a member of your team has scored the winning goal, or even a goal, is immensely satisfying.

I remember going to my first ever Charlton Athletic game. I must note that the crowd was only ever this big when we were doing really well and were in the Championship. Where there were regularly crowds of 25,000 people at some stages. I was really nervous when I entered the ground as to me it was a huge place. We took our seats and I remember feeling a little claustrophobic as there were people all around me with nearly all seats taken. When the game started, the Charlton fans started chanting and I felt it was a really exhilarating experience. When the referee made a decision that did not go our way, I remember the fans shouting a torrent of abuse at the poor referee or one of his two assistants. I calmly looked around and was able to distinguish the fact that the fans were not looking in my direction, they were not interested in me at all, they were only interested in what was occurring on the pitch, and their abusive comments were directed at the referee or one of his assistants and not at me.

I believe I mentioned in a previous blog post that thanks to the AVATAR therapy I received, the voices in my head appeared to sound computerised, I found this enabled me able to distinguish the voices in my head from those around me in the Home fans stand and rationalise that they would not be shouting abuse at me. Why would they be shouting at me when we were supporting the same team? I believe I was able to work all this out thanks to the AVATAR therapy. I must stress that this did not happen overnight and sometimes I have heard the occasional abusive voice at a game. However, the more I persisted in going to the games and concentrating on the football, the more I was able to watch the whole game, which lasts up to 100 minutes. As I became able to get a clear spell lasting that whole time, so my enjoyment of the games increased further. Now I just take every game in my stride and feel very sorry for the poor referees and assistants as some of the abuse they suffer from aggrieved football fans is quite shocking.

I hope this blog has helped people see how AVATAR therapy played such an important role in enabling me to enjoy things which, before, I would have been too worried or scared to do. I know that it is only my individual experience, but I can imagine that the therapy might help other people who suffer from psychosis to broaden their interests and activities and benefit from similar enjoyment which I have found.

*By Nick Hamilton*

**AVATAR2 Creative Workshop Podcast:**

The podcast was co-created by members of the Patient and Public Involvement team and Research workers working on the AVATAR2 therapy trial, in the context of a Creative Workshop series. Together, they reflected on their experience of being involved with PPI work on the AVATAR2 trial and in research, more generally. They bring their reflections to life by sharing some of the creative pieces which have come out of the workshop:

<https://www.avatartherapytrial.com/resources?wix-music-track-id=9711878123451216&wix-music-comp-id=comp-lodxpyr9>

*Co-written, recorded and edited by Sarah Gogan, Amy Grant, Francis Yanga, Oliver Owrid, Stephanie Allan and Leonie Richardson*
