# Supplementary material for: “There’s no us vs. them, it’s just us”: a creative approach to centring lived experience within the AVATAR2 trial
Source: BMC Psychiatry. 2024 Nov 15;24:807. doi: 10.1186/s12888-024-06268-z (PMC11567013; doi:10.1186/s12888-024-06268-z)
Supplement: Supplementary file 2 — Supplementary Material 2. [file 12888_2024_6268_MOESM2_ESM.docx]

**Supplementary Material - Quotes**

**Additional exemplar quotes from WM & TM reflective spaces illustrating key thematic reflections**

| **Theme** | **Additional Quotes** |
| --- | --- |
| **Relationship Building** | “So I kind of felt like I can’t warm up to this research, I want to, but I don’t want to... it has to be like, reciprocated.” **PPI consultant (WM Space)**  “I think I’ve really gotten to know people a lot better through their check-ins, as well, as with things which are shared later on in the workshop” **PPI consultant (WM Space)**  “I think that’s been a big part for me, kind of, I’m feeling like I’m getting to know people as themselves, and as their work selves” **Research Assistant (WM Space)**  “I’ve got an awful lot out of having that within it [AVATAR2], you make friends, but the kind of creative writing side is a bit more relaxing, so it’s easier and you learn so much about everybody else.” **(WM Space)**  “It has felt like a really, really warm space, which is really nice and I think, yeah, I think part of, like, the inclusivity is that everyone will share. It’s not just directed to, let’s say, like research workers sharing or PPI colleagues share [...] it’s just been a nice space where everyone can feel like they can share if they want to.” **(WM Space)**  “There is that sense of validation and understanding and genuine empathy present too, as well as learning” **(WM Space)**  “This space has been so helpful and feeling you know like we, we're building these relationships...It's made me feel more grounded connected to my job and my team and my colleagues.” **(WM Space)**  “I think if you just showed someone the creative workshop, you’d be like this is how we work together.” **(TM Space)** |
| **Personal Development** | “I just didn't have the confidence, so it's kind of really giving me the confidence to feel like, yeah, maybe I could do this” - **PPI Consultant (WM Space)**  “That's something I've really loved and seeing people sort of change.” – **PPI Consultant (WM Space)**  “It's been like across the across the board, just very helpful for my mental health, for like career ambition or like hobbies and leisure and just self expression and talking to family and friends And I think with like work and stuff, hopefully maybe better at presenting as well.” - **PPI Consultant (WM Space)**  “It was great to be doing something which wasn't, you know, all about the trial and you know that it had a bit of a kind of different feel, that it was more perhaps focused on people's lives and learning new things.” - **(TM Space)**  “Real kind of collective warmth and attentiveness and listening and respect for people's stories.” - **(WM Space)**  “That's something I've really loved and seeing people sort of change and I think mainly finding out their inner thoughts and that has been really helpful and help me with my son [who experiences psychosis] as well. When people have written about what they've been through and what they experience, especially with voices and it gives me an insight into my son, which is really helpful.” **- PPI Consultant (WM Space)**  “What [PPI Consultant] said about the confidence, I agree, because if I go into another workplace now, how I would react would be very different because I've built up the confidence. So if I was being bullied again now, I would have the confidence to talk to like a supervisor. And if it was a supervisor that was bullying me, then I'd go above her head and talk to that person. But I would get help for myself, I think the confidence is good. But also I think for personal development I think I have developed my voice as well. I think it links in with confidence.” - **PPI Consultant (WM Space)** |
| **Research Activities** | “The website is just so much enriched by the content. I think in previous websites we've often wondered, is it getting a bit boring like people just going and clicking and it’s the same content? This felt fresh and engaging.” **- Therapy Coordinator (TM Space)**  “So I think there's something about like integrity about kind of having very different perspectives. You know fine grained data match with kind of rich poetic metaphors that are, you know, perhaps more accessible to different folk and very engaging, I think as kind of has been pointed out as well, but something about the integrity and the holistic nature of the PPI.”**(TM Space)**  “`We definitely use the website and people's posts of what avatar therapy was like and what life could be like afterwards” **(TM space)** |
| **Trial Culture** | “Like, we're invited to everything and I think it's that level when we do go to the massive meetings, you know everyone is included and it just feels like everyone's on the same level and everyone's welcomed” - **PPI Consultant** **(WM Space)**  “I think it's sort of had a really helpful creative friction with us, not friction but you know an impact on how we conceive of the work that we do the research; what does different aspects of the research look like? What does lived involvement look like?” - **Therapy Coordinator (TM Space)**  “One thing to mention is just the relinquishing of a little bit of the oversight and control and allowing something to be there. I think that’s had an influence on me and it sounds like [Trial Manager] and other people here. Just how we kind of think about delivering these big projects and what they can involve” **(TM Space)**  “What's needed is space and time and I think that's something I've taken away from all the PPI work on avatar but the creative workshops in particular is what can be done with someone really taking the time and creating a space and then secondly the outputs have really pushed you know in a really positive way the boundaries of what I see are the outputs as of PPI on a trial being like rather than a recruitment number it's a poem” - **Trial Manager (TM Space)**  “It's kind of broadened it out way beyond what I might have thought. If someone had asked me at the beginning of the trial what I'd expect PPI outputs to be it would have looked very different so I've been grateful for that because I think about it so differently now.” - **Therapy Coordinator (TM Space)**  “It's about hearing lived experience and engaging with it but also in a poetic and sometimes humorous and lovely way. And because people can doubt, we can doubt what we're doing, you know, we can feel that is it worth doing what we're doing? And it sort of brings it up so I think one impact has just been really that it's just so encouraging to the wider team so I really thank you for that.” **- Principal Co-Investigator (TM Space)**  It's holistic and about empowerment and you know doing that with integrity and that it's not actually just all about voices. You know it kind of seems there's a lot about the creative workshops and approach to PPI that to me I wonder if I was not part of the team I’d think wow. Like, that's a trial or that's a team that actually really care about people and really care about empowering all members in the work they do.  **- Trial Manager (TM Space)**  “With like the podcast and you know how that’s evolved and I had some anxiety over it all, how on earth is this going to happen? And then suddenly [RA] is like oh, there’s a studio in King’s College. That’s just like, the “yes culture” just keeps going on and on”- **PPI Consultant (WM space)** |
